# One-Stop Device Closure for Ventricular Septal Defect with Atrial Septal Defect Guided by Transesophageal Echocardiography

**DOI:** 10.31083/RCM26279

**Published:** 2025-04-27

**Authors:** Jinghao Song, Yuekun Sun, Huaixue Mi, Shibin Sun, Hongxin Li

**Affiliations:** ^1^Department of Cardiovascular Surgery, The First Affiliated Hospital of Shandong First Medical University and Shandong Provincial Qianfoshan Hospital, Shandong Engineering Research Center for Heart Transplant and Material, 250014 Jinan, Shandong, China

**Keywords:** congenital heart disease, percardiac, perventricular, peratrial, device closure, transesophageal echocardiography

## Abstract

**Background:**

Ventricular septal defect (VSD) with atrial septal defect (ASD) is a common complex congenital heart disease. This study aimed to evaluate the clinical efficacy and safety of transesophageal echocardiography (TEE)-guided percardiac or combined percutaneous techniques for treating VSD with ASD in patients with varying anatomies.

**Methods:**

This retrospective cohort study reviewed 40 cases of VSD with ASD treated in our center from June 2015 to July 2023. Under TEE guidance, peratrial, perventricular, or combined percardiac/percutaneous approaches were used based on the VSD type and secundum-type ASD. Follow-up examinations, including electrocardiography, transthoracic echocardiography, and X-ray, were performed after surgery at 24 hours, 1, 3, 6, and 12 months, and yearly.

**Results:**

All patients underwent surgery successfully (100%), with 24, 5, and 11 patients undergoing simultaneous closure via the peratrial, perventricular, and combined percardiac/percutaneous approaches, respectively. Among them, there were six cases of a mild residual shunt, three instances of a mild tricuspid regurgitation, two cases of a mild aortic valve regurgitation, one case of a mild mitral regurgitation, and three cases of an incomplete right bundle branch block, all observed after VSD closure; all had resolved within 6 months of the operation. The chi-square test showed no significant differences in adverse event rates among the three surgical approaches (χ^2^ = 0.09, *df* = 2, *p* = 0.957). The Friedman test compared the preoperative and postoperative left ventricular end-diastolic diameter for the three approaches, providing *p* < 0.001, *p* = 0.589, and *p* = 0.445, respectively. None of the patients required reoperation during the follow-up period.

**Conclusions:**

Under TEE guidance, using diverse percardiac or combined percutaneous device closure techniques for the one-stop treatment of different types of VSDs combined with ASD is safe, effective, and feasible. These approaches can be performed as a valuable alternative therapy for selected patients.

## 1. Introduction

A ventricular septal defect (VSD) with an atrial septal defect (ASD) is a common type of complex congenital heart disease (CHD). Surgical repair under general anesthesia with cardiopulmonary bypass has been proven effective for treating VSD combined with ASD. Moreover, an ultrasound-guided device closure technique with zero radiation has been developed, in addition to percutaneous intervention guided by digital subtraction angiography [1, 2], which allows for percutaneous and percardiac approaches to closure, offering advantages such as reduced trauma, faster recovery, and higher success rates [3, 4, 5, 6]. However, until recently, fewer reports have been published on percardiac or percutaneous device closures of VSD combined with ASD. Therefore, this study aimed to provide a one-stop closure strategy for patients with VSD combined with ASD guided by transesophageal echocardiography (TEE) via percardiac (peratrial approach, lower mini-sternotomy approach, or left parasternal approach) or combined with percutaneous approaches, and to evaluate its benefits and clinical efficacy.

## 2. Materials and Methods

### 2.1 Research Design and Population 

**Fig. 1.**
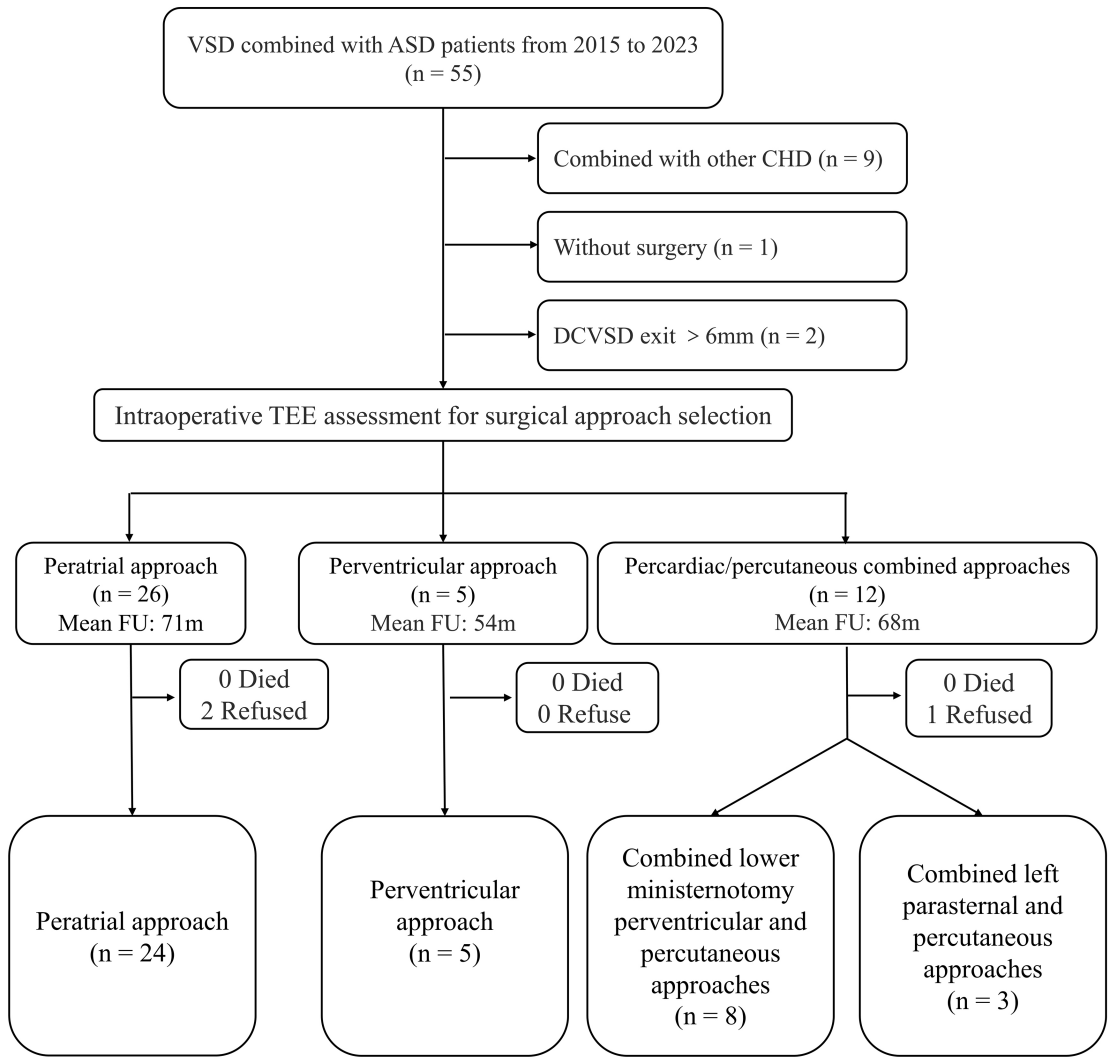
**Flowchart of research design**. TEE, transesophageal echocardiography; FU, follow-up; CHD, congenital heart disease.

This retrospective cohort study was conducted on patients who received a one-stop device closure approach for VSD combined with ASD under TEE guidance at Qianfoshan Hospital between June 2015 and July 2023. All patients underwent either peratrial, perventricular, or combined percardiac/percutaneous approaches for device occlusion (Fig. 1). Our institution obtained baseline data through physical examination, electrocardiography (ECG), transthoracic echocardiography (TTE), and chest X-ray. The anatomical features of defects, the surgical route, and the occluder type were re-evaluated intraoperatively by TTE and TEE.

Among the patients, 24 used the peratrial approach, 5 used the perventricular approach, and 11 used the combined percardiac/percutaneous approaches. The combined percardiac/percutaneous approaches were defined as lower median mini-sternotomy perventricular or left parasternal perventricular approaches for VSD occlusion and percutaneous approaches for ASD occlusion. Informed consent was obtained from each adult patient or the legal guardians of minors. This study was approved by the ethics committee of Qianfoshan Hospital (S747). Data for the baseline characteristics are shown in Table 1.

**Table 1.** **Clinical data of the patients undergoing one-stop device closure for VSD with ASD**.

		Peratrial approach	Perventricular approach	Combined percardiac/percutaneous approaches
		n = 24	n = 5	n = 11
Sex (F/M)		12/12	3/2	6/5
Age (years)		3 (0.58–33)	1.5 (0.33–19)	3.8 (2.5–29)
Weight (kg)		14.3 (8–59)	10.5 (6–50)	19 (13–73.3)
PmVSD (cases)		24	5	7
Multiple mVSD (cases)		0	0	1
DCVSD (cases)		0	0	3
Secundum ASD (cases)		24	5	11
Echocardiography				
	VSD exit (mm)	3 (2.5–6)	2.8 (1.5–10)	2.4 (2–4.5)
	VSD entry (mm)	4.3 (3–12)	5.8 (3–18)	3.8 (2.7–15)
	ASD size (mm)	5 (3–13)	3.5 (2.5–6)	5 (4–8.9)
AV distance (mm)		2 (1–3.5)	1 (0–2.5)	0 (0–2)
ICMT (min)		15.3 ± 12.0	20.1 ± 12.0	27.5 ± 13.4
Procedure time (min)		63.4 ± 18.3	86.8 ± 16.6	85.1 ± 7.1
Device				
	VSD occluder (mm)	11 (8–14)	11 (9–21)	10 (8–16)
	ASD occluder (mm)	18 (14–24)	16 (14–18)	14 (14–20)

Note: F, female; M, male; PmVSD, perimembranous ventricular septal defect; mVSD, muscular ventricular septal defect; DCVSD, doubly committed juxtaarterial ventricular septal defect; VSD, ventricular septal defect; ASD, atrial septal defect; AV distance, the VSD distance to the aortic valve edge; ICMT, intracardiac manipulation time.

### 2.2 Surgical Indications

#### 2.2.1 Inclusion Criteria

(1) TTE/TEE confirmation of VSD combined with ASD; (2) both VSD and ASD had left-to-right shunts without severe pulmonary artery hypertension; (3) age ≥6 months, VSD right-sided opening diameter (exit) ≥2 mm; evidence of recurrent upper respiratory tract infections, congestive heart failure, or left ventricular enlargement in patients younger than 6 months or VSD exit <2 mm; (4) for the aneurysmal type of perimembranous ventricular septal defect (PmVSD), VSD exit ≤12 mm or ≤7 mm in adults and children, respectively; (5) for the non-aneurysmal type of PmVSD: If the VSD distance to the aortic valve edge (AV distance) is ≥2 mm, the left-sided VSD opening (entry) must be ≤10 mm in adults or ≤7 mm in children. If the AV distance is <2 mm, the VSD entry must be ≤7 mm in adults or ≤5 mm in children; (6) doubly committed juxtaarterial ventricular septal defect (DCVSD) exit ≤6 mm; (7) no prolapse of the right coronary cusp or non-coronary cusp; (8) secundum ASD, including double-hole secundum ASD; (9) no coexisting cardiovascular conditions requiring treatment under cardiopulmonary bypass.

#### 2.2.2 Exclusion Criteria

(1) VSD too large, exceeding the scope of the aforementioned indications; (2) evidence of moderate or greater aortic valve prolapse or regurgitation; (3) contraindications to antiplatelet therapy; (4) frequent arrhythmias or severe cyanosis; (5) diagnosed with a right-to-left shunt; (6) New York Heart Association (NYHA) class IV heart failure; (7) suspected of or showing symptoms of infective endocarditis; (8) combination of other cardiac diseases requiring surgery under cardiopulmonary bypass.

### 2.3 Surgical Technique 

#### 2.3.1 Transesophageal Echocardiography, Device, and Delivery System

TEE was performed using the Philips IE33 and EPIQ 7C echocardiography machines (Philips Healthcare, Best, Netherlands) with a 2.0–7.0 MHz frequency transducer. During surgery, TEE was applied to assess the size, position, shape, surrounding structures, presence of a membranous aneurysm, number of exits, and AV distance for each VSD, allowing appropriate occluder selection. TEE also assessed the size and surrounding rims of the ASD.

**Fig. 2.**
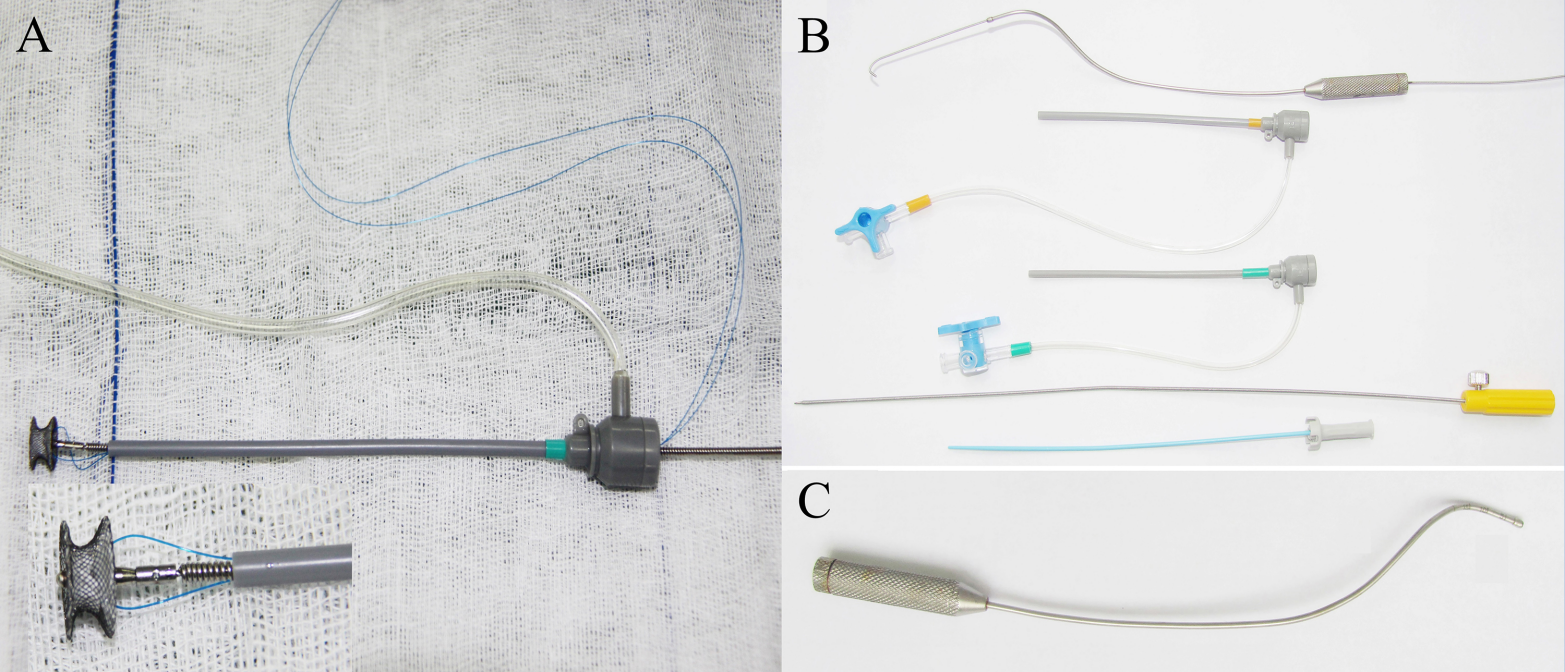
**The delivery systems**. (A) Direct delivery system: A 4-0 or 5-0 polypropylene stay suture was passed through the wire mesh of the selected device beneath the micro screw and then removed from the loading sheath (inset magnified 2.5×). (B) The probe-assisted delivery system. (C) The Z-shaped hollow probe.

The different types of occluders, including VSD occluders (concentric occluder, muscular occluder, and eccentric occluder) and ASD occluders, were supplied by Starway Medical Technology (Beijing, China). The aforementioned company supplied the perventricular and percutaneous delivery systems. The perventricular delivery system employed was the direct delivery system (DDS) and probe-assisted delivery system (PADS) (Fig. 2). The percutaneous delivery system referred to the commonly used digital subtraction angiography-guided interventional delivery system in clinical practice.

#### 2.3.2 Device Selection

For VSD, a concentric occluder 2–3 mm larger than the VSD entry diameter was mostly selected. For PmVSD or DCVSD with an AV distance <1 mm, a concentric short-edged occluder was initially tried; if the device impinged on the aortic valve, it was replaced with an eccentric occluder 2–3 mm larger than the VSD entry. For a small aneurysmal PmVSD, if the VSD exit was a single outlet or multiple clustered exits, a concentric short-edged occluder 2–3 mm larger than the VSD exit diameter was chosen. For a large aneurysmal PmVSD, a concentric wide-edged occluder was selected if the VSD exit was small and the VSD entry was large or had multiple dispersed exits. An occluder size of plus 8 mm is equal to or less than the VSD entry diameter.

For ASD, the device size was selected by adding 4 to 6 mm to the maximum ASD diameter. For a double-hole ASD, the occluder size was determined by adding the larger defect diameter to the distance between the defect edges plus an additional 4 to 6 mm.

### 2.4 Surgical Procedure

#### 2.4.1 Peratrial Approach

**Fig. 3.**
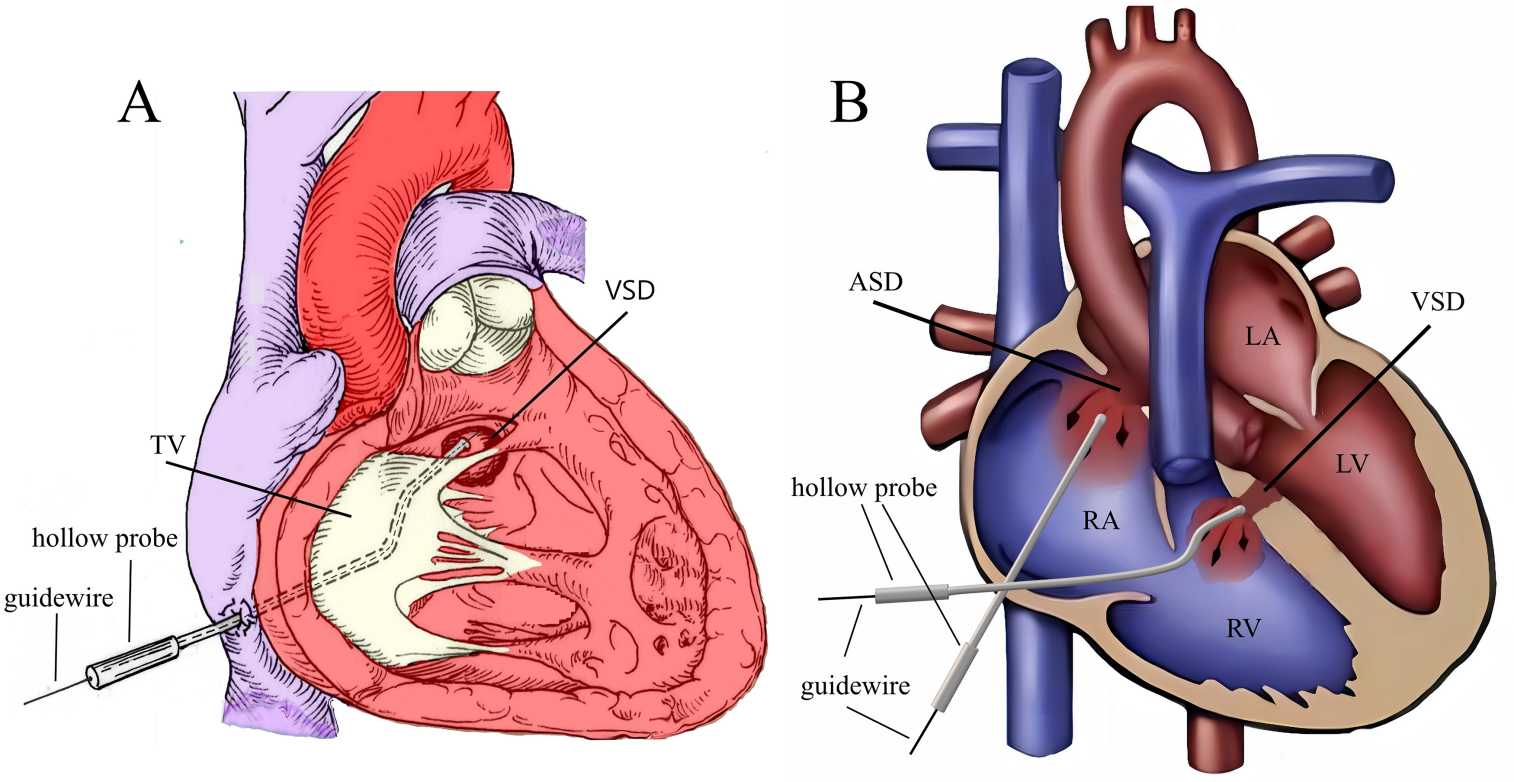
**Schematic of the peratrial approach**. (A) The Z-shaped hollow probe is directed towards the VSD through the TV. (B) First, the Z-shaped hollow probe is directed through the RA, TV, and RV toward the VSD to complete the VSD occlusion. Then, through the same right atrial puncture site, the hollow probe is used to complete the ASD occlusion. Note: RA, right atrium; RV, right ventricle; LA, left atrium; LV, left ventricle; TV, tricuspid valve.

The selected device, connected with a “safety wire” (4-0 or 5-0 polypropylene stay suture), was screwed onto the delivery cable and retracted into the loading sheath. A 1.5 to 3 cm parasternal incision was made in the fourth right intercostal space (within the “bikini lines” in female patients). Superficial tissues were dissected bluntly to enter the pleural space. The pericardium was incised and cradled. Two parallel purse-string sutures of 4-0 or 5-0 polypropylene were placed on the right atrium near the atrioventricular groove. Heparin was administered at a dose of 100 IU/kg. The VSD occluder was then delivered using the PADS. First, the right atrial purse string suture was punctured, and a Z-shaped hollow probe was inserted into the right atrium. Under TEE guidance, the probe tip was advanced through the tricuspid valve into the right ventricle, and the direction of the probe tip was adjusted towards the VSD opening on the right ventricular side (Fig. 3A). Then, a straight, short guide wire was inserted through the probe’s outer hole and across the VSD before the probe was withdrawn. Subsequently, a delivery sheath was introduced along the short guide wire to position it within the left ventricle. The VSD occluder was advanced to the left ventricle, and the left and right disks were deployed sequentially on the respective ventricular sides. Under TEE observation, the position of the occluder was tested repeatedly by pushing and pulling. Once satisfied with its position, shape, and impact on surrounding tissues, the occluder was released. The safety wire was maintained for 5–10 minutes, and a pharmacological blood pressure test (raising systolic pressure to 150 mmHg for adults and 130 mmHg for children) was performed. If satisfied, the safety wire was removed.

After VSD closure, ASD occlusion was performed through the same atrial puncture site. The delivery sheath containing the ASD occluder was inserted through the right atrial purse. The direction of the delivery catheter was adjusted to cross the ASD and enter the left atrium (Fig. 3B). Then, the ASD was occluded. This process does not require a guide wire, and the imaging of the delivery sheath remains clear. Repeated push–pull tests were performed to check the stability of the ASD occluder, residual shunt (RS), and potential impact on the mitral valve and coronary sinus. Once satisfied, the occluder was released.

#### 2.4.2 Perventricular Approach

In the perventricular approach, a 2–3 cm (3–5 cm for adults) incision was made at the lower median sternum near the xiphoid process. The pericardium was incised and cradled to expose the anterior wall of the right ventricle. After heparinization, a peanut dissector was used, under TEE guidance, to gently press the anterior wall of the right ventricle to select a puncture point in the direction of the VSD. A purse-string suture was placed on the right ventricle.

**Fig. 4.**
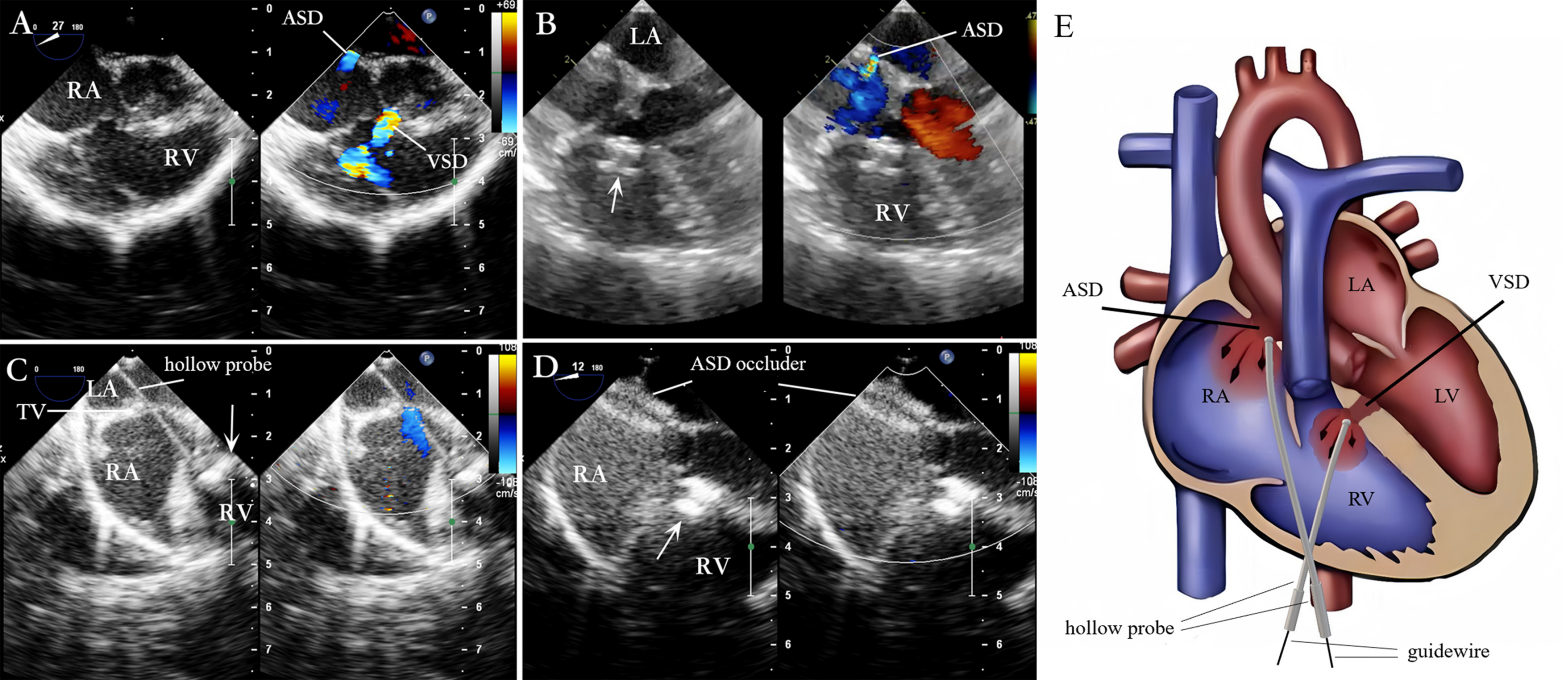
**Lower mini-sternotomy perventricular approach for simultaneous occlusion of VSD combined with ASD**. (A) VSD combined with ASD. (B) After puncturing the RV wall and occluding the VSD, the four-chamber heart view illustrates the ASD. (C) After occluding the VSD, the straight hollow probe passes from the RV through the TV to the RA and is adjusted to pass through the ASD. (D) A guidewire and delivery sheath are inserted along the probe hole to complete the ASD occlusion. (E) The hollow probe is directed towards or through the VSD via the RV puncture site for VSD occlusion. Then, through the same right ventricular puncture site, the hollow probe is directed through the TV into the RA, towards or through the ASD, for ASD occlusion. Note: arrow = VSD occluder.

Under TEE guidance for evaluating VSD and ASD (Fig. 4A), a guidewire, delivery sheath, or hollow probe was used to pass through the VSD. Then, the VSD occlusion was completed (Fig. 4B). Then, the delivery catheter was adjusted, or the hollow probe was re-inserted gently. Next, it was cautiously advanced through the tricuspid valve as it opened to avoid damaging the valve (Fig. 4C). The delivery catheter was passed through the ASD to enter the left atrium before a guidewire was inserted to pass through the ASD into the left atrium; the ASD delivery system was advanced along this guidewire. The occluder was placed to complete the occlusion (Fig. 4D). The position and morphology of the ASD occluder, the RS, and the regurgitation of adjacent valves were evaluated. Once the TEE confirmed the position of the ASD occluder to be satisfactory, the delivery system was removed. The schematic diagram of the procedure is shown in Fig. 4E.

#### 2.4.3 Percardiac/Percutaneous Combined Approaches

(1) Combined lower mini-sternotomy perventricular and percutaneous approaches

VSD and ASD were sequentially occluded using a lower median mini-sternotomy perventricular approach and percutaneous approaches. The steps for VSD occlusion were previously described in 2.4.2 Perventricular Approach section of this paper. After completing the VSD occlusion, ASD occlusion was performed via the right or left femoral vein, following steps similar to traditional interventional methods.

(2) Combined left parasternal–perventricular and percutaneous approaches

**Fig. 5.**
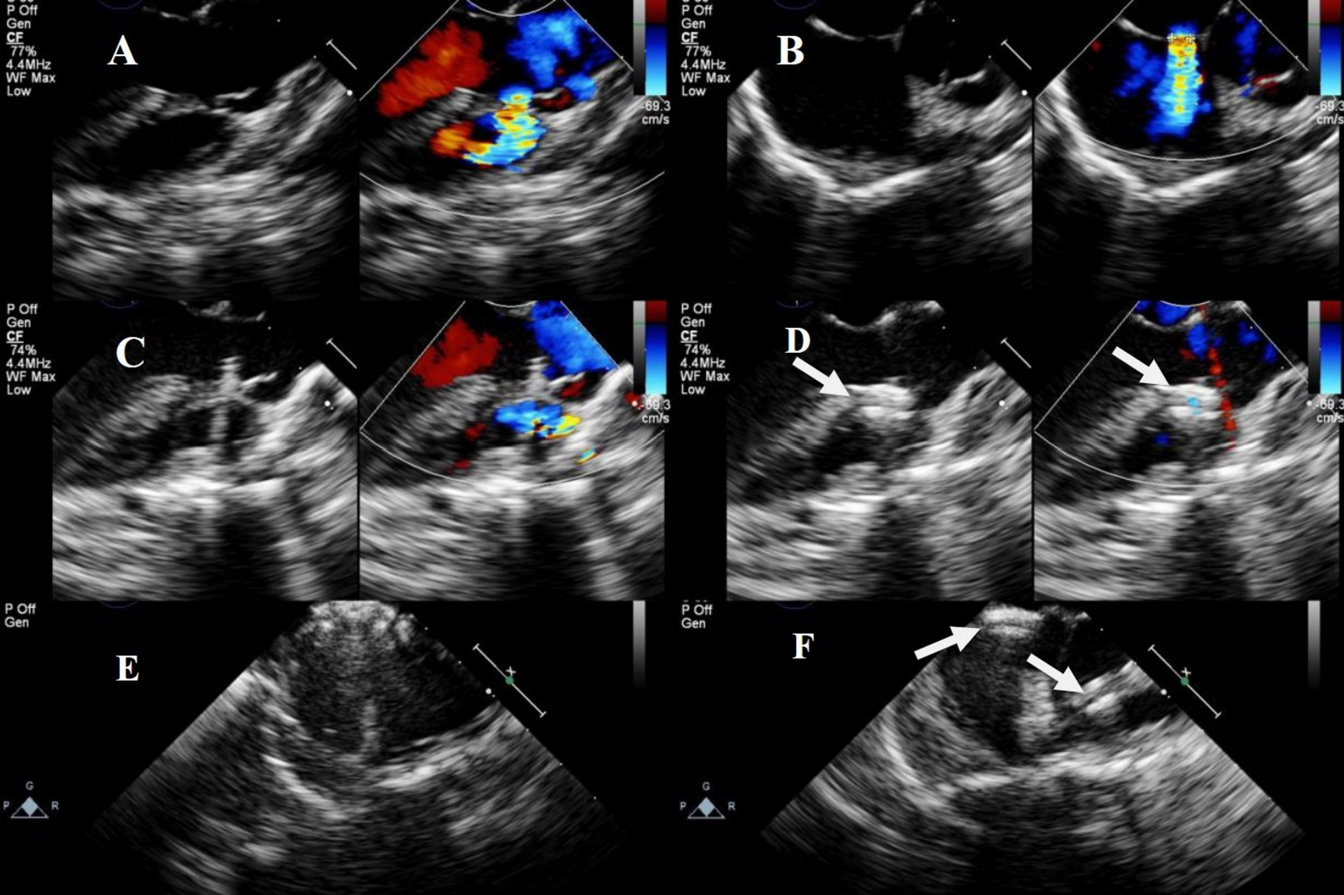
**Transesophageal echocardiography of combined left parasternal–perventricular and percutaneous approaches**. (A) Shunt flow of visible DCVSD. (B) shunt flow of visible ASD. (C) Delivery sheath passing through DCVSD. (D) Successful occlusion of DCVSD. (E) ASD occluded via a catheter. (F) Successful occlusion of both DCVSD and ASD. Note: arrow = occluder.

For DCVSD and PmVSD with a shunt directed towards the pulmonary valve, the left parasternal perventricular approach was used for occlusion, followed by ASD occlusion via the femoral vein. The procedure begins by making a 2–3 cm incision in the second or third intercostal space along the left sternal edge, directing the pericardial incision towards the right ventricular outflow tract. After opening and suspending the pericardium, a puncture point was selected on the surface of the right ventricle, and a double-layer purse-string suture was placed around the puncture point. Then, the right ventricular outflow tract was punctured within the purse-string suture, and the VSD occlusion was completed. Afterward, the ASD occlusion was achieved via the femoral vein route. Fig. 5 illustrates the steps for device closure of a DCVSD with an ASD.

### 2.5 Perioperative Management and Follow-Up

All patients received cephalosporin for infection prophylaxis, which was administered 30 minutes before surgery and continued postoperatively for 48 hours. On the day of surgery, intravenous injections of heparin were administered four times daily after tracheal extubation at 0.2 mg/kg/dose. For antiplatelet therapy, aspirin (3–4 mg/kg/day) was administered orally after tracheal extubation for 6 months to prevent thrombus formation. Postoperative monitoring included an electrocardiogram and pericardial drainage assessment. Most patients were discharged within 4 to 5 days after surgery.

Postoperative follow-up visits were scheduled at 24 hours, 1, 3, 6, and 12 months, with subsequent annual follow-ups. These assessments encompassed physical examinations, TTE, electrocardiogram, and chest X-rays (optional at 6 months). The position and stability of each occluder were meticulously examined, and the presence of an RS and valve-related complications were evaluated at each follow-up.

### 2.6 Data Analysis 

Continuous variables are presented as the mean ± standard deviation or median with an appropriate range, while categorical variables are expressed as frequencies. The differences in left ventricular end-diastolic diameter (LVEDD) among the preoperative, 1-month postoperative, 3-month postoperative, and 6-month postoperative timepoints for the three different surgical approaches (or surgical combinations) were assessed using the Friedman test. A chi-square test was used to evaluate differences in adverse event rates between the three surgical approaches. Statistical analyses were conducted using SPSS 25.0 for Windows (SPSS, Inc., Chicago, IL, USA). A probability value of *p* less than 0.05 was defined as statistically significant.

## 3. Results

### 3.1 Baseline Information and Intraoperative Results

In total, 40 patients underwent successful device placement, implanting 41 VSD occluders and 40 ASD occluders, with a success rate of 100%. Satisfied device placement on the first attempt was achieved in 35 patients (88%); meanwhile, redeployment of the VSD occluders in the same operation occurred in five placements (12%).

Twenty-four patients (12 males and 12 females) underwent peratrial approach closure. All patients had a PmVSD combined with ASD, including two cases of double-hole ASDs. The median age was 3 years (6 months to 33 years), and the median weight was 14.3 kg (8 kg to 59 kg). The VSD entry diameter was 6.29 ± 2.74 mm, the VSD exit diameter was 3.45 ± 1.19 mm, and the maximum ASD diameter was 5.21 ± 3.06 mm. The VSD occluder diameter was 10.26 ± 1.57 mm, and the ASD occluder diameter was 16.74 ± 2.73 mm. Among them, two VSD occluders were replaced with larger ones due to a significant RS. In two patients with double-hole ASDs, a single occluder was used to close both defects.

Five patients (two males and three females) underwent perventricular approach closure. All patients had a PmVSD (including two double-hole PmVSDs) combined with an ASD. The median age was 1.5 years (4 months to 19 years). The median weight was 10.5 kg (6 kg to 50 kg). The VSD entry diameter was 6.38 ± 3.38 mm; the VSD exit diameter was 4.24 ± 2.76 mm. The maximum ASD diameter was 3.84 ± 2.37 mm. The VSD occluder diameter was 12.20 ± 5.02 mm; the ASD occluder diameter was 16.18 ± 1.89 mm. The peratrial approach was initially used in one patient; however, due to the anatomical location of the defect, the guide wire abutted the apex after advancing into the left ventricle. Upon evaluation, the procedure was switched to the lower mini-sternotomy perventricular approach. A single occluder closed both defects in two patients with a double-hole PmVSD.

Eleven patients (five males and six females) underwent percardiac combined with percutaneous approaches closure. Among these patients, three had DCVSD, one had apical multiple muscular ventricular septal defects (mVSDs) (with three holes), and six had PmVSD (with an AV distance of 0 mm), including one with a double-hole PmVSD. All patients had an ASD. The median age was 3.8 years (2.5 years to 29 years), and a median weight of 19 kg (13 kg to 73.3 kg). The VSD entry diameter was 7.70 ± 5.04 mm, the VSD exit diameter was 3.02 ± 1.03 mm, and the maximum ASD diameter was 5.08 ± 1.15 mm. The VSD occluder diameter was 10.19 ± 2.27 mm, and the ASD occluder diameter was 15.75 ± 2.49 mm. In one patient, the device was replaced with a larger eccentric occluder following the compression of the aortic valve, which caused insufficiency. A single occluder was used for one patient with double-hole PmVSD.

**Fig. 6.**
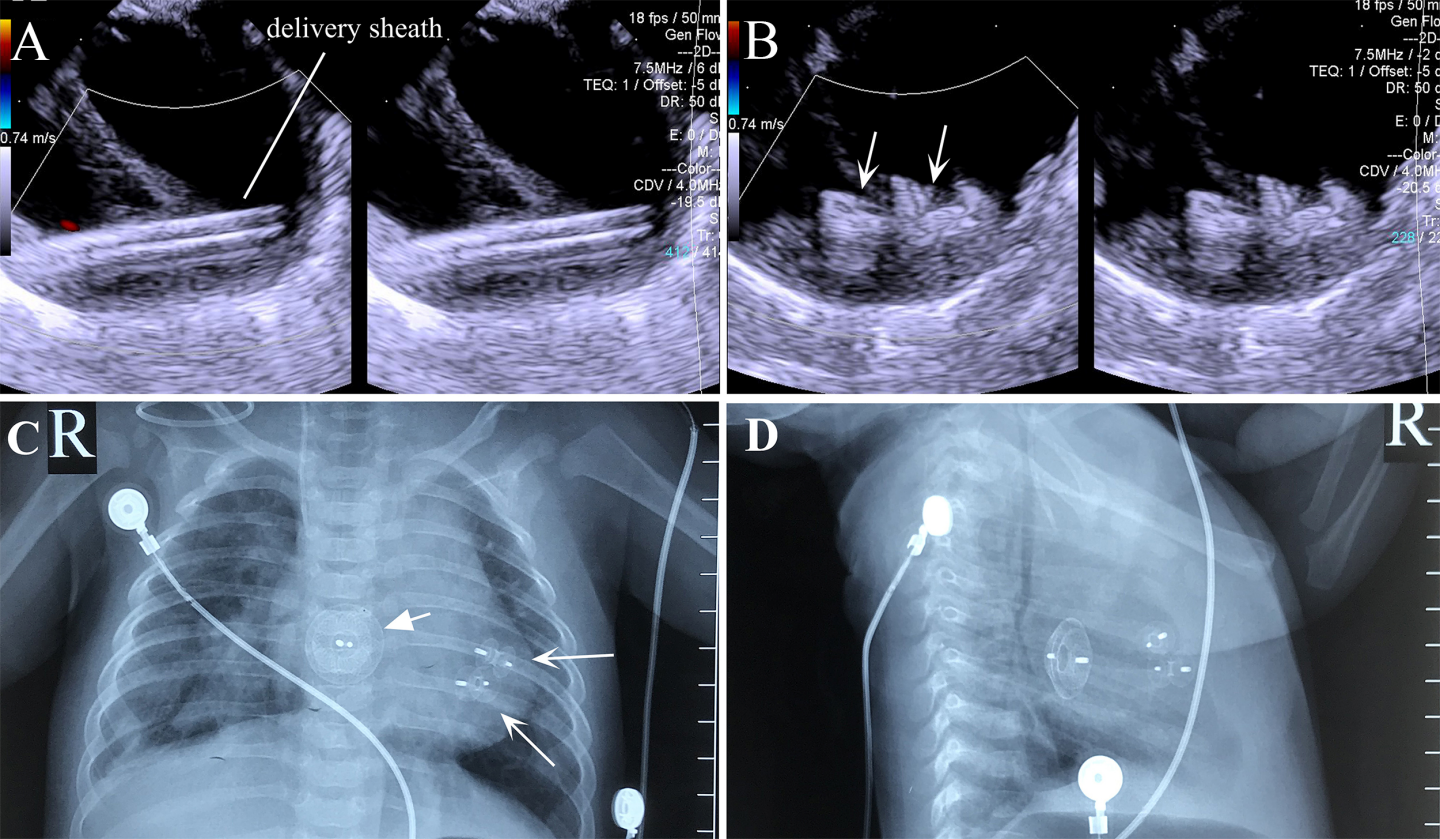
**Transesophageal echocardiography and X-ray images of the combined lower mini-sternotomy perventricular approach for treating multiple mVSDs combined with a percutaneous approach for treating ASD**. (A) Delivery sheath passing through apical mVSD. (B) Successful occlusion of multiple apical mVSDs using two VSD occluders. (C) Postoperative chest frontal view showing two VSD occluders and one ASD occluder. (D) The chest lateral view shows two VSD occluders and one ASD occluder. Note: arrow = occluder.

Two occluders were used during surgery on one patient with apical multiple mVSDs (three holes): a 6 mm waist-diameter muscular occluder was used to close the “middle hole”; meanwhile, the “lower hole” was closed by squeezing the occluder on the atrial septum. However, due to the wide defect spacing and TEE showing a significant shunt in the “upper hole”, a 5 mm waist-diameter muscular occluder was subsequently used to close the “upper hole” (Fig. 6).

### 3.2 Surgery Duration 

The average time from skin incision to skin suturing completion (surgery time) for all patients was 72.85 ± 20.82 minutes. The average surgery times were 63.38 ± 18.34 minutes for the peratrial approach, 86.78 ± 16.64 minutes for the perventricular approach, and 85.05 ± 7.13 minutes for the percardiac/percutaneous approaches. The average time from the entry of the occluder delivery system into the heart cavity to its complete withdrawal (intracardiac operation time) for all patients was 17.72 ± 12.34 minutes. The average intracardiac operation times were 15.29 ± 11.98 minutes for the peratrial approach, 20.14 ± 12.03 minutes for the perventricular approach, and 27.52 ± 13.43 minutes for the percardiac/percutaneous approaches (Table 1).

### 3.3 Follow-Up Results

A follow-up was conducted for 40 patients throughout 16 to 112 months (average follow-up duration of 69.3 ± 29.2 months). Among them, 34 patients had a follow-up period exceeding 18 months, 29 patients had a follow-up period over 36 months, and 18 patients had a follow-up period over 3 years; the follow-up rate was 100%.

Adverse events were experienced by nine patients (37.5%), two patients (40.0%), and four patients (36.4%) in the peratrial, perventricular, and percardiac/percutaneous approaches, respectively. No significant differences in adverse event rates were observed among the three surgical approaches (χ^2^ = 0.09, *df* = 2, *p* = 0.957).

In the peratrial approach (24 patients), four patients (17%) presented with mild RSs immediately after VSD occluder placement, which resolved after one month. One of these patients also exhibited an incomplete right bundle branch block. Three patients (13%) showed incomplete right bundle branch block; two were resolved within one month and the other after one month. Two patients (8%) developed mild tricuspid regurgitation, which resolved within three months. One patient (4%) developed mild aortic valve regurgitation, which resolved after one month. The comparison of preoperative and postoperative LVEDD showed a reduction from 36.0 ± 8.0 mm preoperatively to 34.6 ± 6.2 mm at six months postoperatively (*p* < 0.001).

In the perventricular approach (five patients), one patient (20%) presented with a mild RS, which resolved after three months. One patient (20%) experienced transient sinus bradycardia twice, immediately and 24 hours after surgery; both episodes were controlled using medication. The preoperative and postoperative LVEDD comparison showed a reduction from 30.7 ± 7.5 mm preoperatively to 30.1 ± 5.5 mm at six months postoperatively (*p* = 0.589).

In the percardiac/percutaneous approaches (11 patients), one patient (9%) experienced a mild RS, one (9%) had mild tricuspid regurgitation, and one (9%) had mild aortic valve regurgitation. The conditions of all three patients were resolved after six months. Additionally, another patient (9%) developed mild tricuspid regurgitation, which resolved within one month. The preoperative and postoperative LVEDD comparison showed a reduction from 40.7 ± 10.1 mm preoperatively to 39.8 ± 9.1 mm at six months postoperatively (*p* = 0.445) (Table 2).

**Table 2.** **Follow-up data**.

		Preoperative	24 hours	1 month	3 months	6 months	12 months	3 years	After 3 years	*p*-value
		n = 40	n = 40	n = 40	n = 40	n = 40	n = 34	n = 29	n = 18	
Peratrial approach										
	RS	-	4	2	0	0	0	0	0	-
	New TR	-	2	2	1	0	0	0	0	-
	New AR	-	1	0	0	0	0	0	0	-
	New IRBBB	-	3	1	0	0	0	0	0	-
	LVEDD	36.0 ± 8.0	-	33.5 ± 7.1	34.0 ± 6.1	34.6 ± 6.2	-	-	-	<0.001
Perventricular approach										
	RS	-	1	1	1	0	0	0	0	-
	Bradycardia	-	1	0	0	0	0	0	0	-
	LVEDD	30.7 ± 7.5	-	29.1 ± 7.0	29.4 ± 5.5	30.1 ± 5.5	-	-	-	0.589
Combined percardiac/percutaneous approaches										
	RS	-	1	1	1	1	0	0	0	-
	New TR	-	1	0	0	0	0	0	0	-
	New AR	-	1	1	1	1	0	0	0	-
	New MR	-	1	1	1	1	0	0	0	-
	LVEDD	40.7 ± 10.1	-	39.9 ± 10.5	40.1 ± 9.8	39.8 ± 9.1	-	-	-	0.445

Note: RS, residual shunt; TR, tricuspid regurgitation; AR, aortic regurgitation; MR, mitral regurgitation; IRBBB, incomplete right bundle branch block; LVEDD, left ventricular end-diastolic diameter.

## 4. Discussion

Currently, surgery with cardiopulmonary bypass support remains the most effective strategy for treating VSD with ASD. However, due to issues such as extensive trauma, large scars, and prolonged postoperative hospitalization, an increasing number of surgeons are turning to research on minimally invasive interventional surgical methods [7, 8, 9, 10, 11, 12].

Recently, percutaneous intervention techniques have demonstrated high success rates and low complication rates in treating isolated VSD or ASD [13, 14, 15]. However, this technique has certain limitations depending on the age, weight, and specific anatomical characteristics of the patient and may not be suitable for all types of cardiac defects.

Specifically, for treating PmVSD, patients typically need to be at least 2 years old, weigh ≥10 kg, and have an AV distance of ≥2 mm [15, 16, 17]. When treating ASD, percutaneous intervention techniques require an ASD edge distance from the coronary sinus, superior and inferior vena cava, and pulmonary vein openings to be at least 5 mm and a distance from the atrioventricular valve of at least 7 mm [18, 19, 20]. Considering these issues, various minimally invasive surgical methods developed in recent years have expanded the scope of surgical applications [7, 8, 12, 21]. Among them, the TEE-guided minimally invasive percardiac closure technique has been widely used and has become one of the primary methods for isolated CHD.

For VSD combined with ASD patients, especially infants, early intervention may be required due to the potential severe pulmonary congestion. Although percutaneous intervention techniques have been widely applied, they may not be the safest and most effective solution for certain cases, such as those with DCVSD, mVSD at the apex, a large VSD that is not easily occluded, or those associated with mild aortic valve prolapse. With an increase in the number and duration of catheter insertions, there is a corresponding increase in the risks of atrioventricular block, procedural time, and radiation dose [11, 22, 23, 24]. Additionally, younger patients may face challenges in using intervention catheters due to limitations in their vascular access [23, 24].

**Fig. 7.**
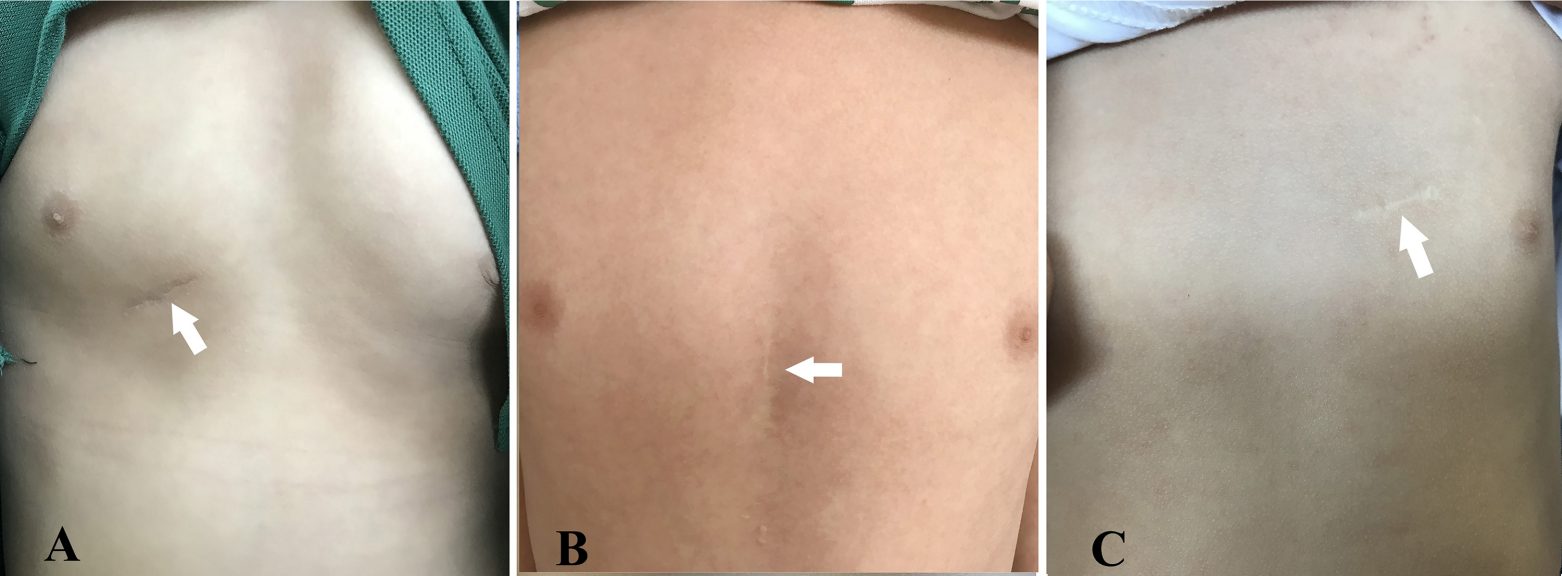
**Photographs demonstrate that postoperative scars have significantly faded over several years**. (A) Right chest small incision (peratrial approach). (B) Lower sternotomy small incision (lower mini-sternotomy perventricular approach). (C) Left parasternal small incision (left parasternal–perventricular approach). Note: the arrow in each panel indicates the postoperative scar.

To date, there are fewer reports on percardiac closure for VSDs combined with ASDs [25, 26]. For percardiac closure of a VSD combined with an ASD, our methods contain the peratrial approach, lower mini-sternotomy perventricular approach, and left parasternal–perventricular approaches. For moderately sized PmVSDs with favorable edge conditions and a shunt direction towards the right atrium and the anterior wall of the right ventricle, our center uses the peratrial approach with a 1.5–3 cm incision (Fig. 7A) through the right chest to the right atrium for VSDs. This method resulted in satisfactory therapeutic outcomes and follow-up results without impairing right ventricular function. For PmVSDs that are too small (<2 mm) or too large (>7 mm), with an AV distance less than 2 mm, without a membranous aneurysm, multiple defects, or with shunt streams close to the septum, and apical mVSDs, the peratrial approach for VSD closure may be relatively challenging. Due to the favorable orientation of the right ventricular approach for eccentric positioning of the occluder relative to the aortic valve and apex, our center tends to select a device closure procedure through a lower mini-sternotomy perventricular approach for such patients (Fig. 7B). Meanwhile, for patients with DCVSDs, the perventricular approach may make achieving the necessary perpendicular angle to the defect difficult unless the lower mini-sternotomy incision is extended upward [27]. In such cases, our center tends to choose the left parasternal–perventricular approach (Fig. 7C).

The main approaches for ASDs are percutaneous and transthoracic device occlusions. For patients with an ASD who are at least 2 years old and weigh at least 10 kg, if the defect margins are suitable for occlusion (i.e., the distance between the ASD edge and the coronary sinus, superior vena cava, inferior vena cava, and pulmonary vein is at least 5 mm, and the distance from the atrioventricular valve is at least 7 mm, with an ASD diameter of less than 25 mm), the first option is percutaneous approach. However, for ASD patients with multiple defects or a short distance to the superior or inferior vena cava edges, or for ASD who are younger than 2 years old or weigh less than 10 kg, if they have severe symptoms or right ventricular volume overload, the peratrial approach may be safer [7]. In short, if the ASD is too large, numerous, or has poor edge conditions, the peratrial approach is chosen; if the ASD is small with favorable edge conditions, the percutaneous approach is preferred.

Due to the anatomical characteristics of VSDs, completely avoiding the RS immediately after surgery is difficult. In this study, the RS of a VSD were all approximately 1 mm and completely disappeared during follow-up. The VSD occluder may affect the function of the aortic and tricuspid valves, while the ASD occluder may affect the mitral valve, leading to regurgitation. The peratrial approach for VSD occlusion and the perventricular approach for ASD occlusion involves passing the delivery system through the tricuspid valve, which may increase the risk of valve injury. If moderate or greater regurgitation develops, immediate device replacement or adjustment should be considered. In this study, three patients experienced mild tricuspid regurgitation, while two cases of mild aortic valve regurgitation were noted, and one patient developed mild mitral regurgitation postoperatively; all disappeared during the follow-up. The most common arrhythmia is a newly developed incomplete or complete right bundle branch block after occluder implantation. In this study, incomplete right bundle branch block developed in 3 of 40 patients postoperatively and disappeared within 3 months of follow-up. One patient who underwent the perventricular approach experienced two episodes of transient sinus bradycardia postoperatively within 24 hours, which did not recur before discharge or during subsequent follow-up, likely related to vagal nerve stimulation. Gentle intraoperative handling, selecting an appropriate occluder, and avoiding excessive repeated attempts during the operation could help reduce the incidence of complications.

The percardiac/percutaneous approaches under TEE guidance do not require digital subtraction angiography or contrast agents, reducing equipment needs and costs, and the technique is easy to master. Furthermore, the percardiac approach is not restricted by age, weight, or vascular conditions, has a shorter delivery path for the occluder, and offers more convenient operation and greater flexibility in surgical planning. However, these approaches are not suitable for all types of VSD or ASD. Specifically, for complex cases, traditional surgery is still considered the safest treatment option to ensure patient safety and surgical outcomes. Despite the advantages of TEE, this technique necessitates general anesthesia and tracheal intubation for assisted breathing. The percardiac approach also carries risks such as scarring, pleural effusion, pneumothorax, sternal deformity, or bleeding.

### Limitations

While our results are encouraging, our study has limitations. Firstly, the sample size is small, as only 40 patients were included in this study. Future studies should include more patients to demonstrate the safety and effectiveness of this technology. Secondly, for patients with a VSD combined with an ASD, this study did not include patients who underwent staged device closure surgery. We could not compare patients who underwent staged or one-stop device closure surgery to demonstrate the advantages of one-stop surgery. Thirdly, it is a single-center study: multicenter studies with longer follow-up periods are needed to evaluate the future long-term effectiveness and complications of the described techniques.

## 5. Conclusions 

This study confirms that under TEE guidance, selecting different approach combinations for one-stop device closure of a VSD combined with an ASD is safe and feasible. This treatment plan, which applies different combinations of device closure techniques based on patient-specific conditions, does not increase the risk of complications and ensures a high success rate. Tailoring the surgical approach to the specific anatomical conditions of VSDs and ASDs in each patient, utilizing optimized techniques such as percardiac or combined percutaneous device closure under TEE guidance, offers several advantages: patient-specific treatment, high success rate, minimal trauma, small scars, no radiation exposure, and broad applicability, thereby providing new perspectives and valuable reference for clinical practice.

## Data Availability

The datasets used and/or analyzed during the current study are available from the corresponding author on reasonable request.

## Abbreviations

CHD, Congenital Heart Disease; TTE, Transthoracic Echocardiography; VSD, Ventricular Septal Defect; ASD, Atrial Septal Defect; PmVSD, Perimembranous Ventricular Septal Defect; mVSD, Muscular Ventricular Septal Defect; DCVSD, Doubly Committed Juxtaarterial Ventricular Septal Defect; DDS, Direct Delivery System; PADS, Probe-assisted Delivery System; RS, Residual Shunt; LVEDD, Left Ventricular End-Diastolic Diameter.
